# Analysis of Hospitalization Length of Stay and Total Charges for Patients with Drug Abuse Comorbidity

**DOI:** 10.7759/cureus.6516

**Published:** 2019-12-30

**Authors:** Memory Ndanga, Shankar Srinivasan

**Affiliations:** 1 Health Information Management, Rutgers University, Piscataway, USA; 2 Informatics, Rutgers University, Piscataway, USA

**Keywords:** drug abuse, comorbidity, length of stay, national inpatient sample, cost, inpatient days, drug misuse, drug dependence, hospitalization, total charges

## Abstract

Background

Drug abuse has been on the increase over the last few years, contributing to the healthcare cost. An understanding of the overall impact of drug abuse hospitalizations is essential in combatting the drug abuse epidemic.

Objective

To evaluate inpatient outcomes of total charges and length of stay for patients with drug abuse comorbidity compared to non-drug abuse admissions.

Method

The Healthcare Cost and Utilization Project (HCUP) Nationwide Inpatient Sample data was utilized. Drug abuse comorbidity was used as defined by HCUP. Various descriptive and inferential analyses were performed on the filtered data sets for the years 2010 to 2014.

Results

The average hospitalization length of stay was 4.5 days for non-drug abuse and 5.5 days for drug abuse comorbidity (P < 0.001). Mean charges for drug abuse comorbidity were significant for claims to private insurance and Medicaid.

Conclusion

Total charges and length of stay are higher for drug abuse than non-drug abuse cases. The results will aid as a reference for resource allocation and policy changes. Further research is needed for alternative and innovative interventions for conditions that are identified to be co-existing with drug abuse comorbidity.

## Introduction

Drug abuse has increased in the past two decades, causing a substantial burden not only to the families but also to the healthcare system and the United States (US) economy as a whole. This study analyzes the outcome of the length of stay and total charges of patients with drug abuse comorbidity. Studies on drug abuse comorbidity are of importance in resource allocation and can be used as a necessary reference tool primarily as the nation is geared towards combatting the current drug abuse problem. The majority of people know someone with a substance use disorder or someone who has lost a relative or friend due to substance misuse. Drug abuse has become more prevalent, such that it is not a surprise that there were 27 million adult Americans who have self-reported the misuse of illegal drugs or opioid-based prescription drugs in 2015 [1]. The drug abuse hospitalization cost was over $21 billion in 2010 and is expected to grow as the epidemic continues. The business sector has also identified the second largest expense for large and small businesses, apart from salary, being health care for their employees. Productivity losses related to personal and family health problems is estimated to cost US employers $1,685 per employee per year, or $225.8 billion annually [2], of which drug abuse is a contributing factor. The total healthcare expenditure for 2014 was set to have increased by 4.1% [3]. Hospital admissions of drug-related diagnoses are potentially avoidable, saving the healthcare system a substantial amount of money and resources [4-5]. The Agency for Healthcare Research and Quality (AHRQ) reported that the national rate of drug abuse-related inpatient stays and emergency department (ED) visits increased 64.1% and 99.4%, respectively, in 2005 - 2014 [6]. Therefore, there is a need to analyze the drug abuse comorbidity pattern.

In a cost comparison study that was initiated to calculate the excess burden of drug abuse, the drug abuse group had higher utilization rates for medical services and prescription drugs. The study identified patients with drug abuse using medical and pharmacy claims data. The results were compared with non-opioid abuse diagnosis using linear regression. Drug abusers had an eight times higher cost than non-abusers. Hospital inpatient visits for opioid abusers were 12 times higher than non-abusers, resulting in an estimated mean annual cost of at least $15, 884 compared to $1,830, respectively [7]. At least one in every five days was attributed to substance abuse stay, and 1.2 million days were used for direct treatment of substance abuse, i.e., treating diseases wholly attributable to substance abuse. With the constant increase in medical costs, policies are focusing on reducing resource consumption by decreasing the number of inpatient days [8-9]. Marijuana studies also examined the incremental inpatient costs of treatment that showed that marijuana comorbidity was associated with longer length of stays and higher charges [10].

## Materials and methods

For this project, data was taken from the National Inpatient Sample (NIS), which is part of the Healthcare Cost and Utilization Project (HCUP). HCUP is associated with the AHRQ. HCUP combines both private and state organization data, including the Federal government. It is the most extensive collection of longitudinal hospital data. All-payer and encounter levels information is also provided. Researchers have been able to utilize this database for research on a broad range of healthcare policies, including qualities on the cost of services, access to healthcare and programs, and outcomes of treatment at all levels.

This study includes comorbidity drug abuse cases from the 2010 - 2014 HCUP National Inpatient Sample Database [11]. The design for this quantitative approach included a thorough analysis of hospitalization outcomes for the selected years. The IBM Statistical Package for Social Sciences (SPSS) (IBM SPSS Statistics, Armonk, NY) and Microsoft® Excel (Microsoft® Corp., Redmond, WA) were used in the analysis and preparation of the data. Drug abuse comorbidity cases were filtered for data analysis. Various descriptive and inferential analysis were performed on the filtered data sets for the above years.

## Results

**Figure 1:**
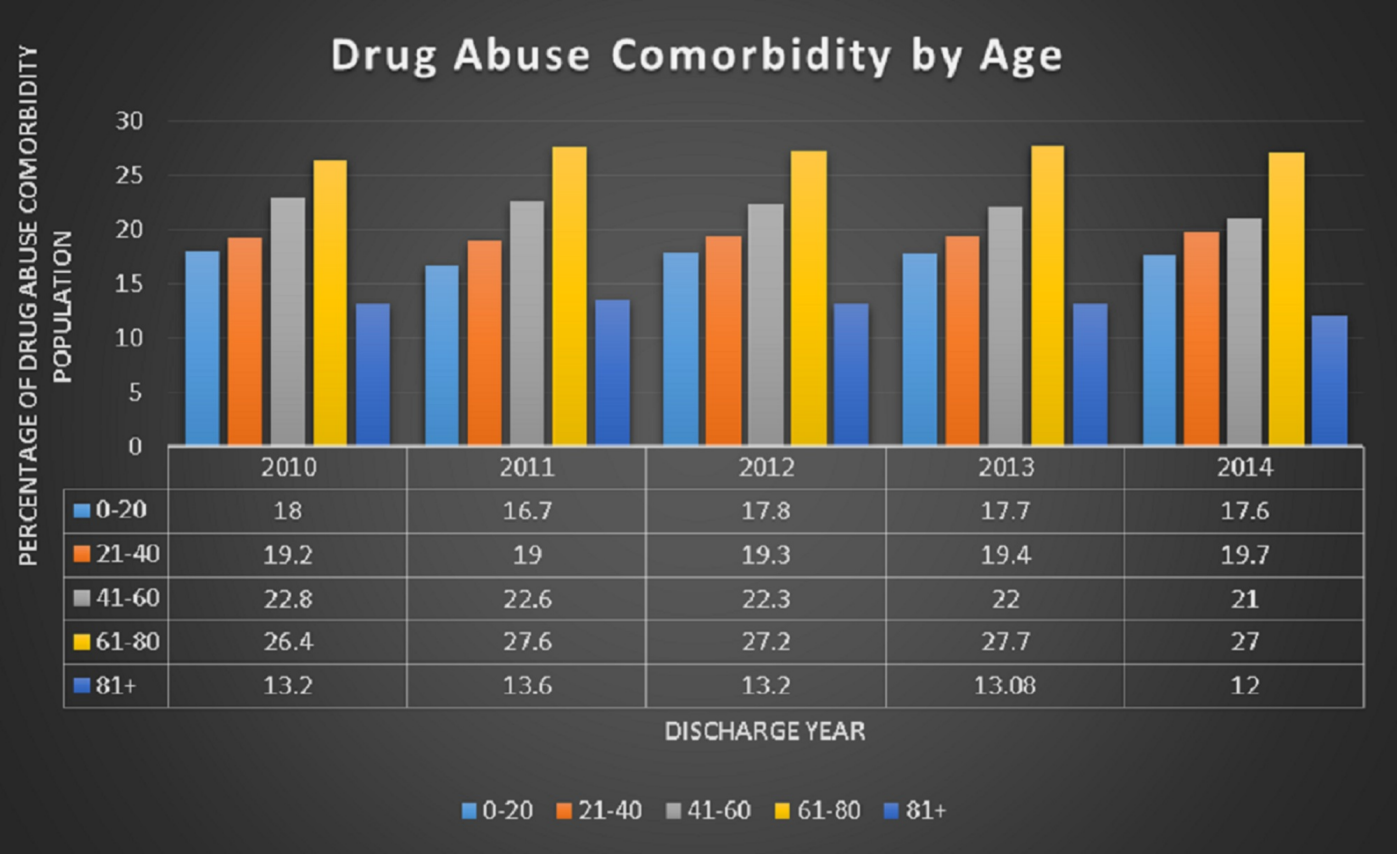
Age groups of drug abuse cases CM: comorbidity

The study sample included a total of 2,258,235 drug abuse comorbidity patients, of which 54% were males and 46% were females (p < 0.001). The age range for patients with drug abuse comorbidity varied, as shown in Figure 1 below. In order to better understand the differences in age groups, the patient ages were divided into five groups ranging from 0 - 81 and older as shown in Figure 1. The mean age of the study population was 44.45 with a standard deviation (SD) of 6.72 years (mean age for females was 43 years, while males were 45 years, p < 0.05). 

**Figure 2:**
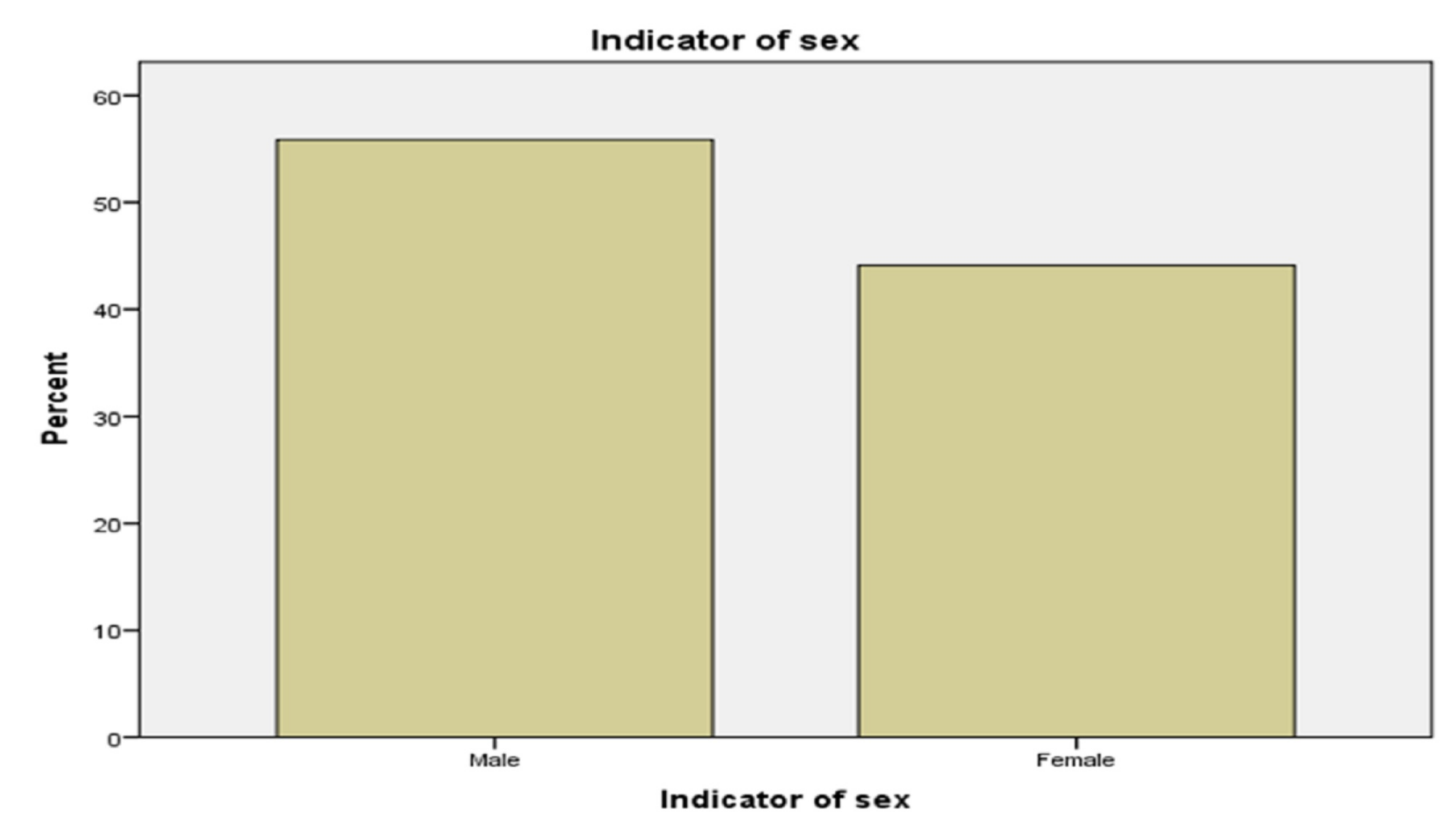
Gender and Drug Abuse Comorbidity

Figure 2 shows the gender difference for patients with drug abuse comorbidity; 54% were males and 46% were females (p < 0.001).

**Figure 3:**
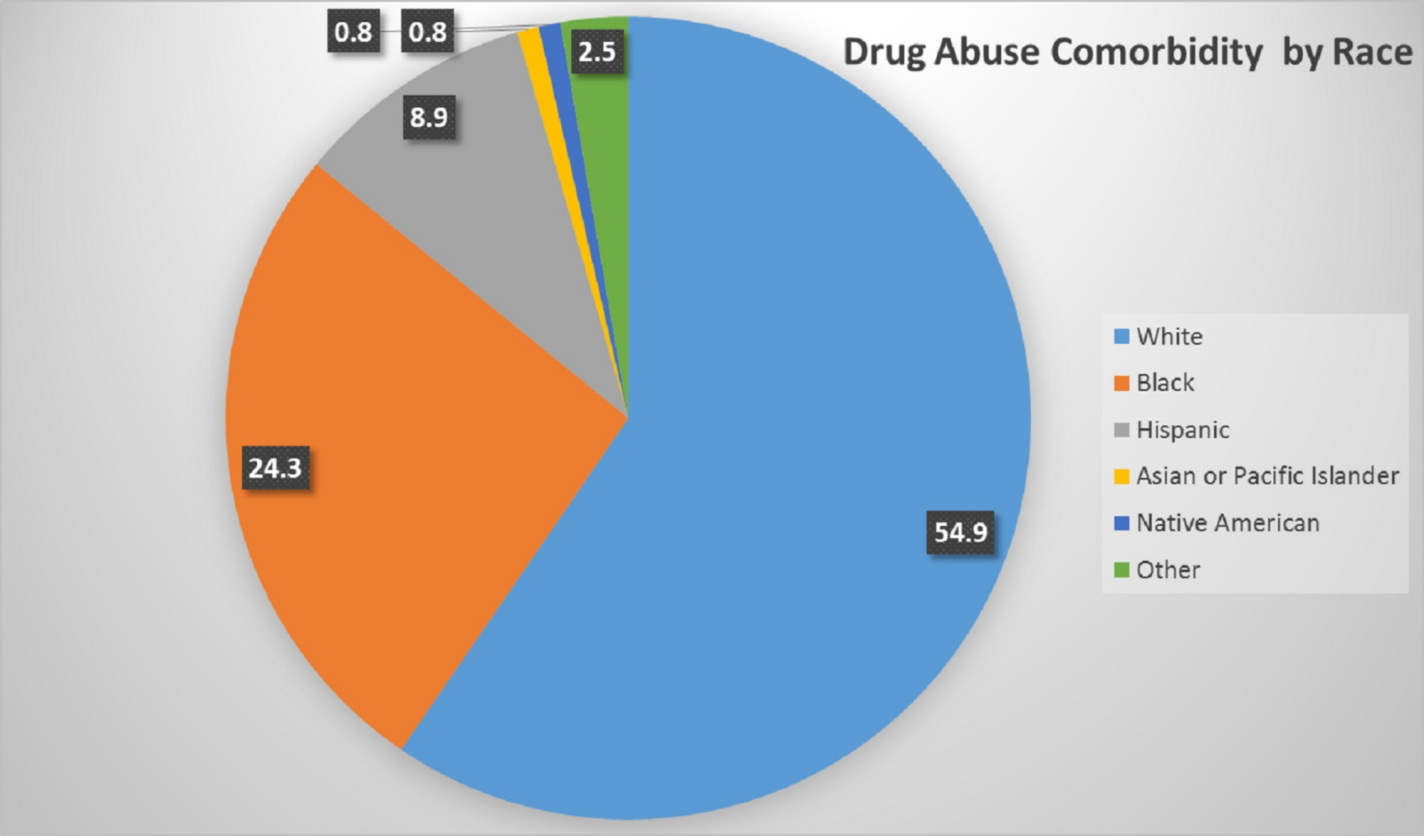
Drug Abuse Comorbidity and Race Comparison

A total of 629,608 drug abuse comorbidity patients were White, which comprised 54.9% of the total, followed by Black (24.3%), Hispanic (8.9%), Asian (0.8%), Native American (0.8%), and others (2.5%). The race distribution is shown in Figure 3.

Table 1 shows the primary expected payer and that Medicaid was the main form of health insurance charged 36%. Medicare had 22.6% while private and self-pay had 18.2 and 15.1, respectively and other not specified insurance types were charged 5.4%.

**Table 1:** Shows Primary Expected Payer

		Frequency	Percent
Valid	Medicare	510,179	22.6
	Medicaid	827,936	36.7
	Private	411,272	18.2
	Self-pay	341,257	15.1
	No Charge	37,369	1.7
	Other	122,357	5.4
Total		2,258,235	100

When comparing the length of stay for between drug abuse comorbidity patient and non-drug abuse, the average stay for a patient with drug abuse comorbidity was 5.52 compared to 4.56 for non-abuse as shown in Table 2.

**Table 2:** Length of Stay Comparison for Comorbidity Drug Abuse and Non-drug Abuse SD: standard deviation

Drug abuse	Mean	Number	SD
Comorbidity Drug Abuse Not Present	4.56	286,979,236	6.768
Comorbidity Drug Abuse Present	5.52	11,211,054	8.051
Total	4.59	298,190,290	6.823

**Figure 4:**
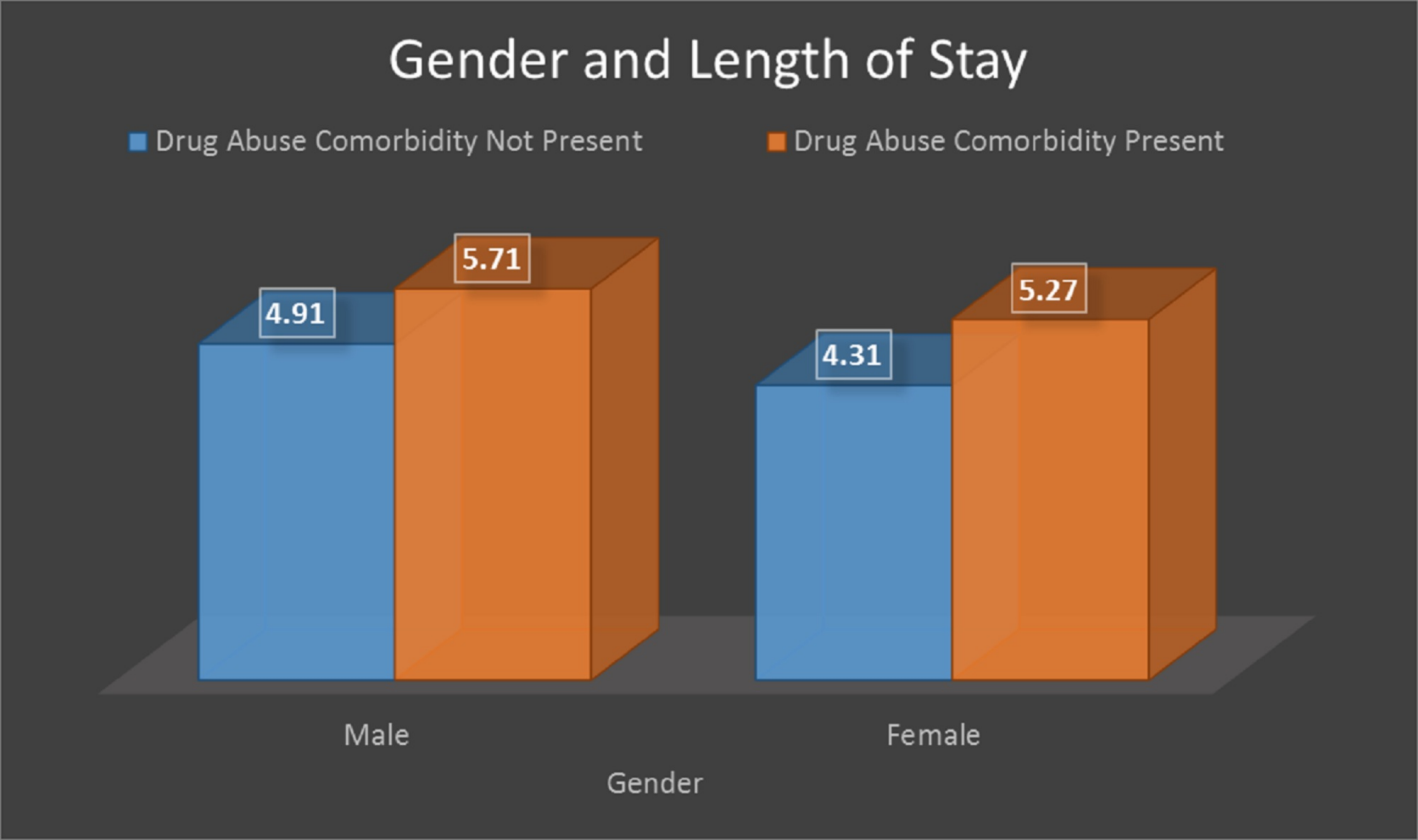
Length of stay comparison by gender

The length of stay comparison by gender is shown in Figure 4 reflects that males with drug abuse comorbidity had an average of 5.7 inpatient days, while male non-drug abuse patients stayed for 4.9 days. Females who had drug abuse comorbidity had an average of 5.2 days, while female non-drug abuse patients had 4.3 inpatient days. The p-value was significant at p < 0.001

**Figure 5:**
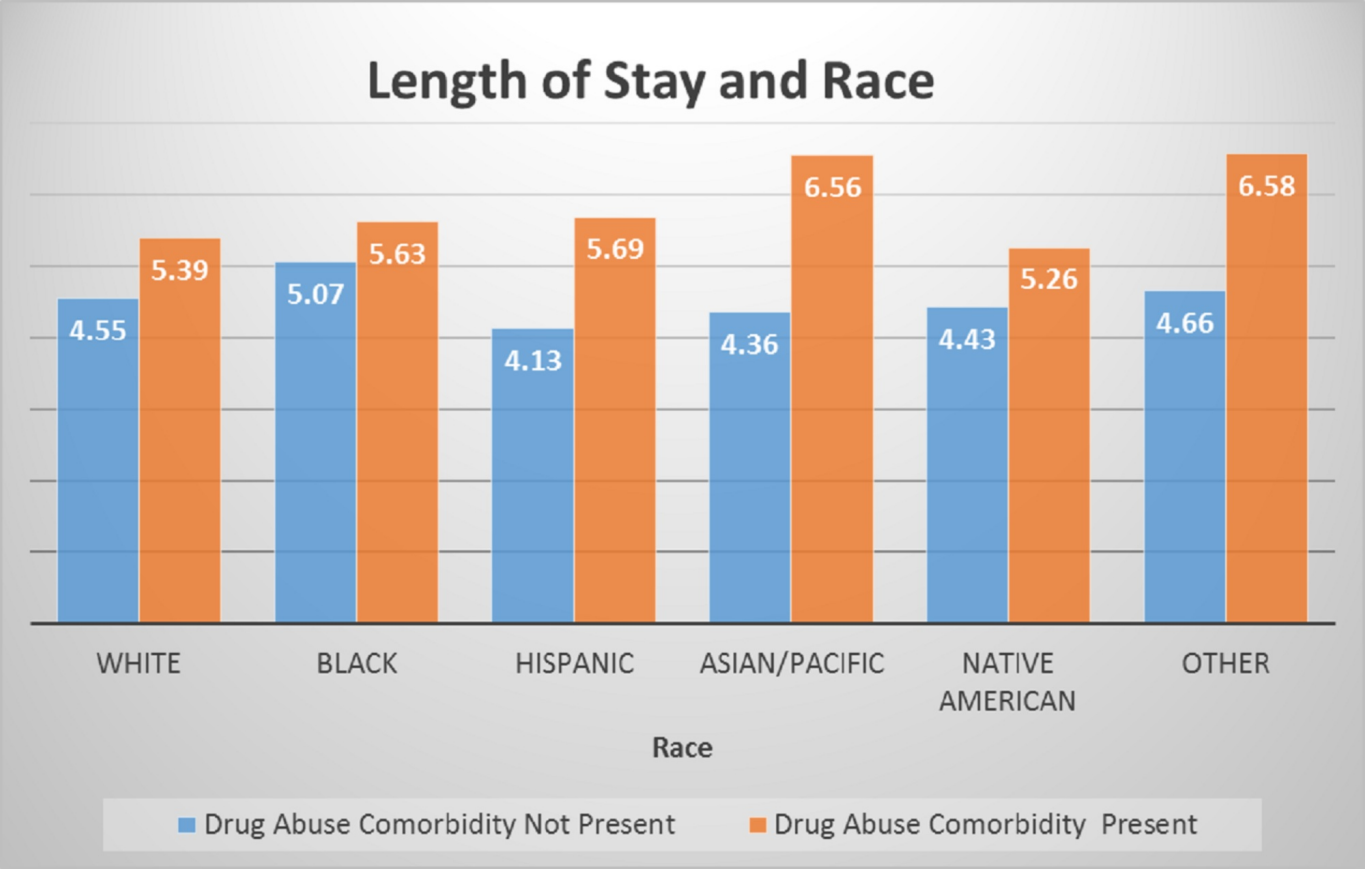
Length of stay comparison by race

The length of stay comparison between races shown in Figure 5 varied. The range of the inpatients days were 4.1 - 4.6 days for non-drug abuse cases, while drug abuse comorbidity ranged from 5.2 - 6.5 days, as shown in Figure 5. The p-value for race and length of stay was significant at p < 0.001 and a 95% confidence interval.

**Figure 6:**
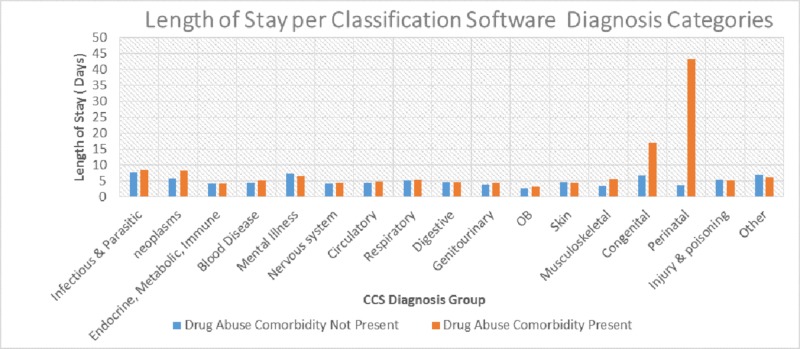
Length of stay comparison by diagnosis categories CCS: Clinical Classifications Software; OB: obstetrics

Classification software diagnosis categories were used to identify the relationship between the length of stay and diagnosis categories. Figure 6 shows that the lowest inpatient days were recorded for obstetrics non-drug abuse of 2.6 days and 3.1 for drug abuse comorbidity. The most extended stay for non-drug abuse was 7.6 days for the infectious category, while the highest for drug abuse was for a perinatal category that had a total inpatient stay of 43.2 days.

**Figure 7:**
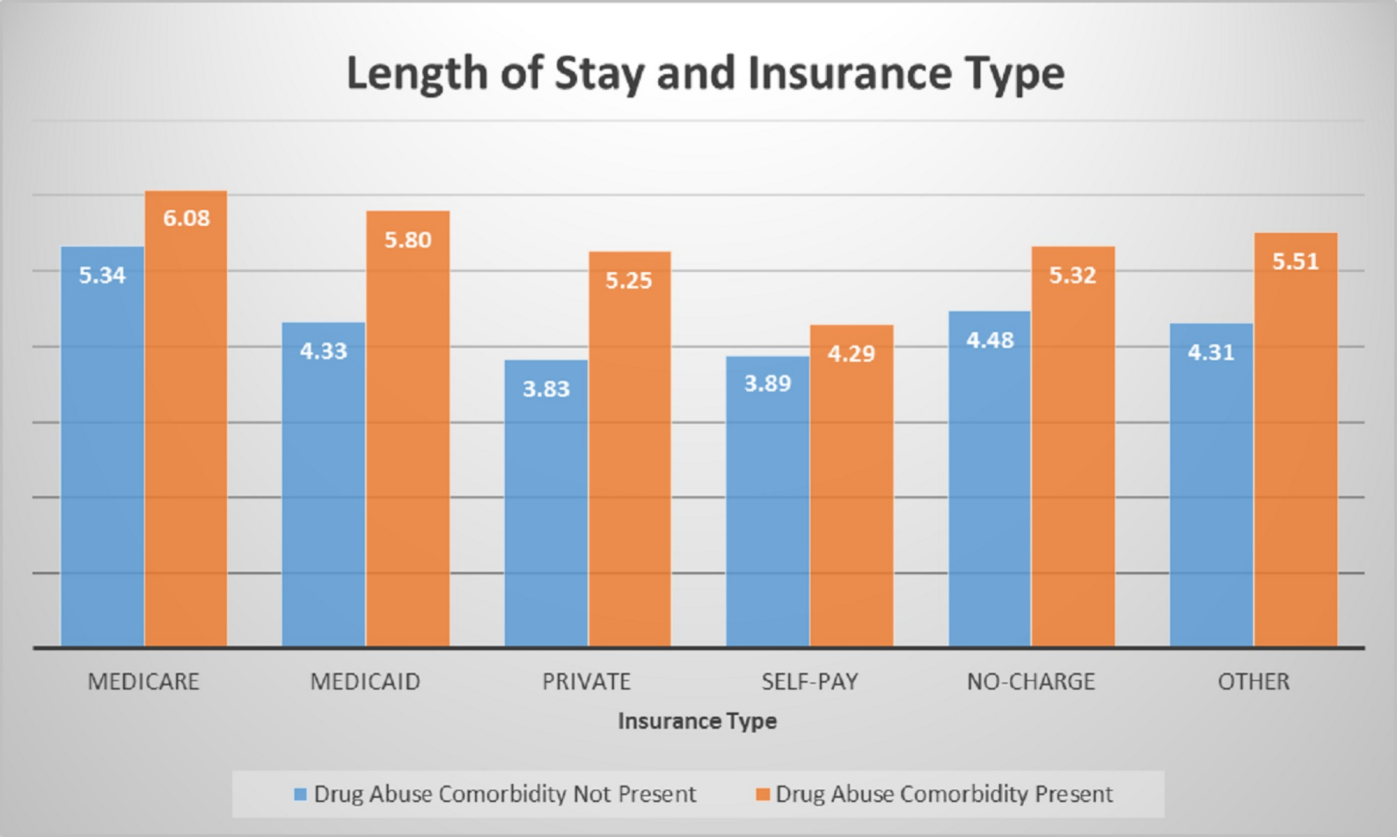
Length of stay comparison by insurance type Significant p-values < 0.05 at 95% confidence interval

Figure 7 shows the association of the length of stay and type of insurance. Medicare patients with drug abuse comorbidity spent six days compared to five days for non-drug abuse. The same trend was seen for all insurance types that insurance companies have to pay for a day more for drug abuse comorbidity patients compared to non-drug abuse.

**Figure 8:**
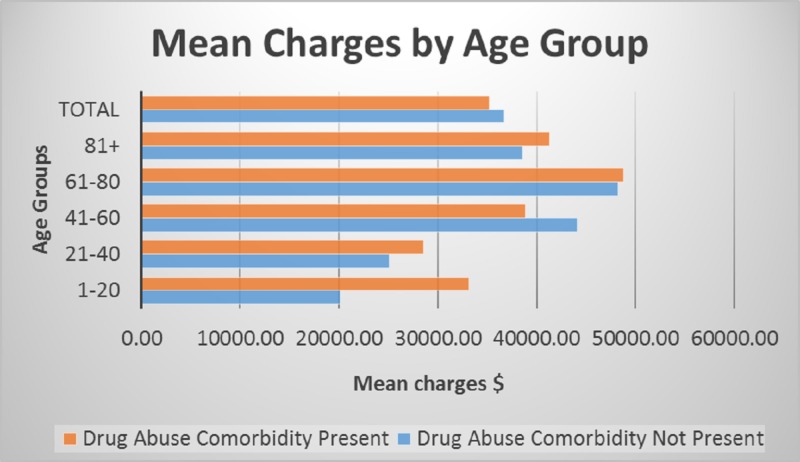
Hospital charges comparison by age group

The mean charge for each age group shown in Figure 8. The 0 - 20-year-old age group of non-drug abuse cases had $20,000 in charges, and the drug abuse comorbidity cases had $34,000. The 21 - 40-year-old age group had $25,000 for non-drug abuse, while the drug abuse comorbidity had $28,000. The 41 - 60-year-old age group had $44,000 for non-drug abuse, while the drug abuse group had $38,000. Older adults aged 61 - 80 years had $48,000 for drug abuse comorbidity and the non-drug abuse group had $47,000. Lastly, 81 years old and above had $42,000 drug abuse comorbidity and $38,000 for non-drug abuse cases. The results were significant at p < 0.001 and 95% confidence interval.

**Figure 9:**
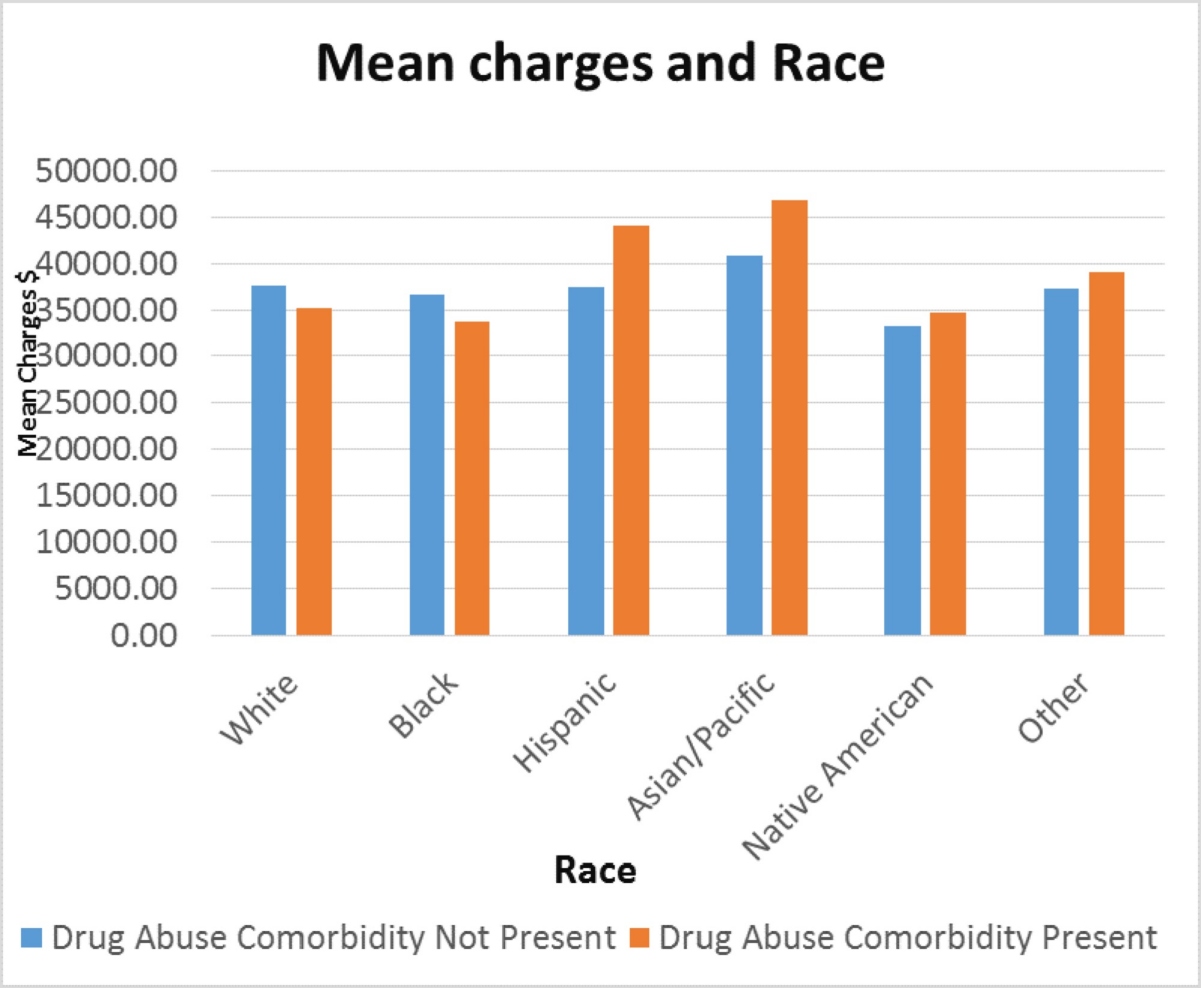
Comparison of hospital mean charges by race

The mean charges for race in Figure 9 show that for non-drug abuse, Caucasians had $38,000 and $35,000 for the drug abuse group. Blacks had $36,000 for non-drug abuse group, while the drug abuse comorbidity group had $34,000. Hispanics showed $44,500 for the drug abuse comorbidity group and less than $40,000 for non-drug abuse group. The Asian/Pacific Islander race showed higher charges at $47,000 for the drug abuse comorbidity group and $41,000 for the non-drug abuse group. Native Americans and Others, not specified race had $34,000 and $39,000, respectively, for the drug abuse comorbidity group and the non-drug abuse group had $33,000 and $36,000, respectively. There was a significant P-value of less than 0.001.

**Figure 10:**
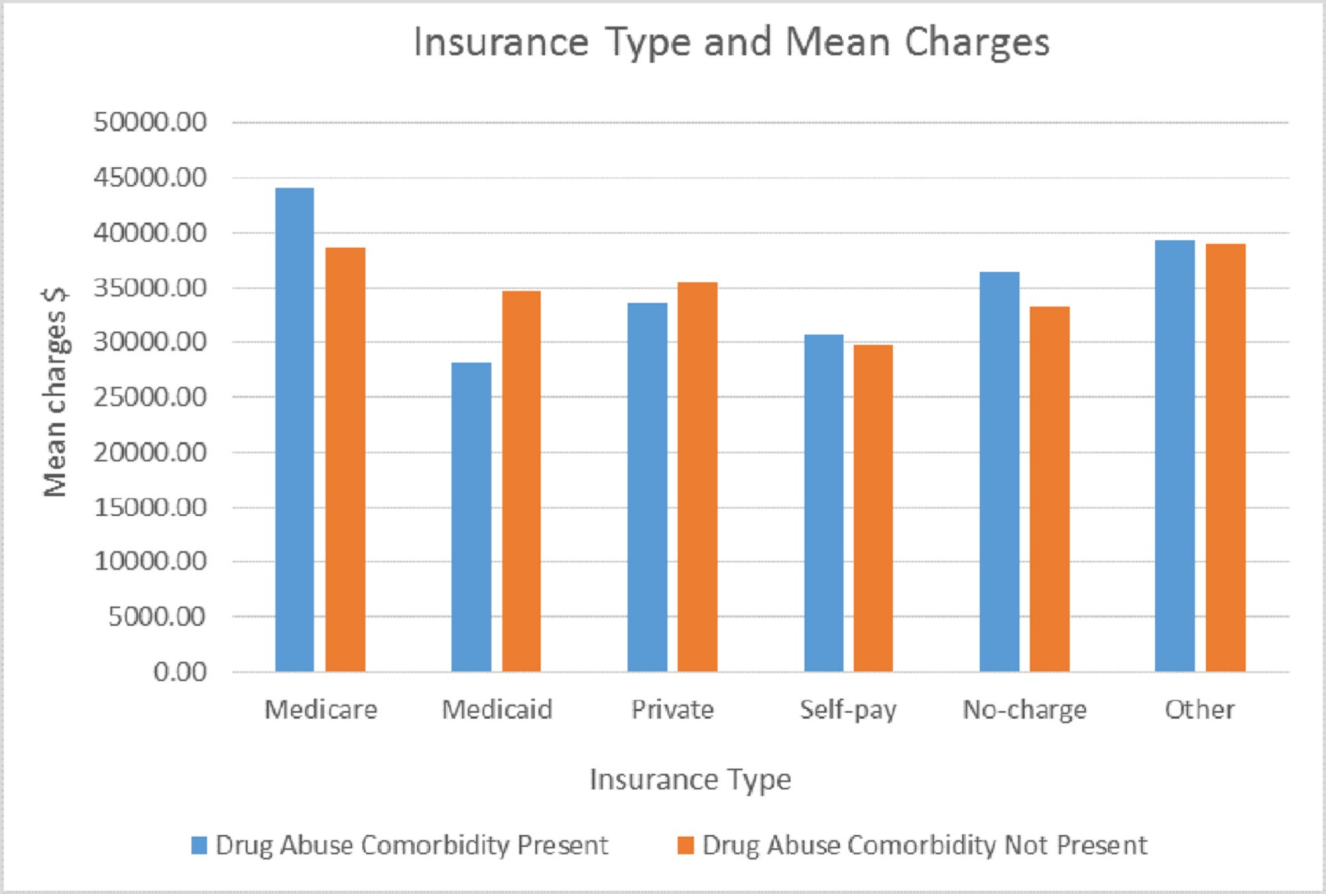
Comparison of total charges and insurance type

Figure 10 shows Medicaid and private insurance paid more for drug abuse comorbidity than non-drug abuse cases. Medicaid recorded charges for patients with drug abuse comorbidity at $35,000 compared to non-drug abuse charges of $26,500 for the same insurance type. Private insurance also showed significant charges for drug abuse comorbidity cases at $36,000 compared to non-drug abuse cases at $33,450. 

## Discussion

In this retrospective study, we demonstrated that most of the drug abuse comorbidity patients were Caucasian males who were 44 years of age. These findings support the findings of other publications that have indicated that men are more likely than women to use almost all types of illicit drugs [12]. Men, in general, have a higher rate of drug and alcohol use compared to women [13]. We found substantial differences between races; Caucasians recorded the highest number of patients admitted with drug abuse comorbidity at 54%, while Blacks and Hispanics had 24% and 9%, respectively. All other races had less than 10% of the patients that were admitted with drug abuse comorbidity. In other studies that have looked at the different types of drugs in trauma settings, Caucasian and Hispanic patients were more likely to be admitted [14-15]. Blacks in rural areas were less likely to use methamphetamine compared to whites [16-17]. This is probably because each race is likely susceptible to a different type of drug, hence the need to expand the research on the types of drugs affecting each race. The US population surveys and treatment studies also indicated that there is a racial and ethnic difference in the prevalence of substance use [18]. There is also evidence that racial differences also result in minorities less likely to receive a variety of medical services. These disparities are significant even when looking at insurance status, income, age, and education level [19] and are more intensified when drug abuse and other mental disorders are present [20].

The most significant percentages of drug abuse-related stays were for patients within the two age groups, 41 - 60 and 61 - 80, which had an average of 22% and 27%, respectively. Although older adults might not typically be considered drug seekers, it is likely that as their primary or secondary conditions become more clinically complicated, the continuous use of prescription medication might lead to addiction misuse or dependence. Older adults are also fragile and are susceptible to multiple factors. Other studies have also considered the potential of drug interactions associated with multiple conditions [21-22].

Maternal drug use also contributed to cases of babies that were less than two years to be included in the drug abuse population. Drug abuse comorbidity for infants is more costly and complicated. Substantial progress for the baby that has been exposed to drugs is very complicated and is attributed to the longer length of stay [11, 23].

It is also necessary that healthcare providers should consider the complete medical and social factors contributing to healthcare as abuse intensifies existing conditions [24-25]. A higher proportion of patients with drug abuse comorbidity showed that the most prevalent principal diagnosis was depression and mood disorders. Other forms of psychosis were also noted to be very common in patients with drug abuse comorbidity. This correlates with other previous studies that have identified a similar pattern of co-occurrence of substance-related disorders and personality or mood disorders [26-27].

The need for prophylactic vaccination was also another common diagnosis for a patient with drug abuse comorbidity compared to non-drug abuse. This is possible to prevent infections related to injection drug use. Injection site studies have identified the increase in drug use-related infections and attributed them to a significant mortality and morbidity rate and a substantial hospitalization cost [28].

The mean length of stay was five days for both males and females. The longer the substance abuse patients were admitted, the more resources they were likely to use. An article by Heslin et al. found an association between specific drug abuse diagnoses with longer lengths of stay [29]. Table 2 shows that the mean hospital length of stay was a day longer in drug abuse comorbidity patients compared to non-drug abuse patients (4.56 versus 5.52 days with a p-value < 0.00). An additional day of inpatient care increases resource utilization and adds to the overall cost of hospitalization. A more comprehensive and intervention targeted for drug abuse-related diagnosis could potentially reduce the length of stay. The Clinical Classifications Software (CCS) diagnosis category analysis showed that perinatal and congenital categories have an extremely higher length of stay when drug abuse comorbidity is present (43.2 days and 16.8 days, respectively) compared to 6.7 and 3.7 days, respectively, when there is no drug use. Respiratory and hemic/lymphatic procedures also showed a longer length of stay when drug abuse comorbidity was present (12 and 11 days, respectively) compared to general non-abuse (nine days).

The study demonstrates the contribution of drug abuse comorbidity to the overall health service utilization. There was a significant difference in total charges for cases that reflected drug abuse comorbidity compared to non-drug abuse. When identifying charges for each age group, there were variations in total charges for each variable of interest. Total charges for the group aged 1 - 20 years were high for drug abuse comorbidity cases ($33,000) compared to the non-drug abuse group ($20,000). The young adults aged 21 - 40 showed higher charges for drug abuse comorbidity cases with $28,100 compared to $24,600 for non-drug abuse. The older adults had mean charges of $38,000 for non-drug abuse patients compared to $42,000 for the drug abuse comorbidity patients.

The mean total charges, according to race, also showed that Hispanics and Asian/Pacific Islanders had higher hospital charges when they had a drug abuse comorbidity on record, even though they had fewer cases of drug abuse comorbidity admissions. This could be due to cultural differences and hesitation in seeking immediate treatment for their condition. However, when they do seek treatment, the treatment charges are likely to be higher because of being admitted through the emergency department or for treatment for intoxication and inpatient stays, resulting in additional hospitalization costs. Lack of insurance coverage has been linked to limited access to healthcare resources for minorities which is of significant concern as Hispanics, Blacks, and some Asian/Pacific Islander populations appear to have lower levels of healthcare coverage compared to Caucasians [30].

The primary health insurance for the sample admissions was mostly either Medicaid, private insurance, or those without health insurance which probably could also be that the health insurance was not listed during the time of discharge. Medicaid covered more than 36% of the total patients admitted with drug abuse, followed by Medicare 23%. This reflects that there is a significant burden on government-related health insurance benefits. This also possibly indicates the socioeconomic background of drug abuse patients. Medicare charges are the highest for drug abuse cases compared to general hospitalizations, which is in correlation with other Medicare and Medicaid cost studies [30].

To effectively provide information on charges and length of stay for drug abuse comorbidity, the Clinical Classification Software (CCS) for the International Classification of Diseases, Ninth Revision, Clinical Modification (ICD-9-CM) diagnosis and procedure categorization scheme was used. CCS categories have over 14,000 diagnosis codes and 3,900 procedure codes, which are collapsed into a smaller number of clinically meaningful categories that are more useful for presenting descriptive statistics than are individual ICD-9-CM codes. The CCS for ICD-9-CM, a diagnosis and procedure categorization analysis of charges, shows that certain conditions, such as perinatal and congenital diagnoses, will increase charges significantly when associated with drug abuse comorbidity compared to non-drug abuse cases. All CCS procedure categories also reflected an increase in charges when drug abuse comorbidity was present, suggesting the need for innovative interventions that are focused on addressing diagnosis and procedures that are coexisting with drug abuse comorbidity. Drug abuse comorbidity contributes to higher charges; therefore, linking the directly related diagnosis can provide a care plan that will reduce resource utilization and cost and improve patient clinical outcomes.

Our results depend on accurate coding of the diagnosis using the ICD-9-CM. The administrative database is an excellent resource for healthcare research, but as with any data, it is subject to coding inaccuracies and deficiencies. The lack of detailed information on patient progress during inpatient care limits the understanding of the length of stay causes, including readmission rates. The lack of cost breakdown on proportionality on total charges or costs limits the ability to stratify costs further. Despite these limitations, the data source is a large and nationally representative database that allows information to be captured from a majority of admissions and discharges in the US. The data includes all regions and covers a significant number of factors that allow a generalizable analysis of all hospitalizations in the US.

## Conclusions

This study brings an understanding of the hospitalization outcomes of drug abuse comorbidity patients. We identified that charges are higher for drug abuse comorbidity than general admissions, drug abuse comorbidity hospitalization cases have a longer length of stay than non-drug abuse cases, and there are variations in age, race, and insurance payer for drug abuse comorbidity and non-drug abuse hospitalizations. Patients with drug abuse comorbidity have been noted to have other conditions that have resulted in a costlier inpatient stay, suggesting a more targeted approach for patient care for those who fall under the CCS diagnosis category of perinatal, congenital, and mood and personality disorders. Hospital mean charges for government-related insurances is also of the importance to policymakers who can evaluate potential costs and estimate budgets. Continuous research is needed to understand substance abuse comorbidity hospitalization in a more comprehensive approach, including identifying alternative and innovative interventions for conditions that are identified to be coexisting with drug abuse comorbidity. 
